# College Students’ Reflections on Their Experience Facilitating a Photovoice Research Project with BIPOC Older Adults and Frontline Healthcare Workers

**DOI:** 10.3390/youth3020033

**Published:** 2023-03-31

**Authors:** Angela U. Ekwonye, Tenzin Chonyi, Iqra Farah, Stephanie Nguyen, Abigail Malek, Mary Hearst

**Affiliations:** 1 Department of Public Health, St. Catherine University, St. Paul, MN 55105, USA; 2 School of Nursing, University of Minnesota, Minneapolis, MN 55455, USA

**Keywords:** college students, photovoice research project, reflections, BIPOC older adults, BIPOC frontline healthcare workers

## Abstract

The photovoice literature has focused mainly on research collaborations between researchers in academia and community partners. There is limited evidence of undergraduate students facilitating photovoice research projects with underserved Black, Indigenous, and persons of color (BIPOC). Students’ participation in photovoice research increases their understanding of local issues and can empower them to engage with local communities to create change. This qualitative study explored students’ experiences facilitating photovoice research with BIPOC older adults and frontline healthcare workers. In this study, we trained college students in photovoice methodological skills by introducing photovoice as a participatory qualitative methodology, explained ethics issues and the informed consent process, and clarified the steps and requirements for the photovoice project. Students were paired with BIPOC older adults and frontline healthcare workers. They facilitated the photovoice research project in four stages. Throughout the study, students critically reflected and wrote about the various aspects of their experience facilitating the photovoice research activities based on weekly structured reflection questions. In their weekly journal, student facilitators commented on how both older and younger BIPOC participants were friendly and kind, flexible with the research activity scheduling, inspiring with their stories, and selfless. Students’ reflections indicated that their participants experienced greater emotional and psychological burdens during the COVID-19 pandemic, exacerbated by the long-standing epidemic of systemic racism. Student facilitators overwhelmingly recognized participants’ resilience and perseverance despite these life challenges. Our findings highlight the importance of photovoice in developing and strengthening college students’ qualitative research skills and stimulating critical thinking and creativity, a sense of critical consciousness about social issues and society, and a sense of empowerment. Our results will be relevant to hospital/long-term care managers, policymakers, and researchers.

## 1. Introduction

This article discusses the reflections of college students who facilitated a photovoice research project with BIPOC older adults and frontline healthcare workers. Through a description of the project, a report of the research findings, and a discussion of the project’s impacts, this study provides evidence of the power of photovoice as means for students to critically examine their knowledge and perception of the lived experiences of BIPOC older adults and frontline healthcare workers during the COVID-19 pandemic. Photovoice has been described as having three primary objectives: (1) to enable people to record and reflect on the strengths and concerns of their community, (2) to promote critical dialogue and knowledge about important issues through large and small group discussions of photographs, and (3) to reach policymakers [1]. Like other forms of community-based participatory research (CBPR), photovoice offers opportunities to engage individuals that typically would not have the experience, skill-set, or confidence to conduct research [2]. Photovoice can foster greater participatory engagement between students and subject matter, knowledge, and learning [3]. Reflections from students who participated in a photovoice food security research revealed that students expanded their understandings of the local issues and imagined their roles in addressing those issues in future professional practices [4]. Photovoice can also play a role in youth development and leadership by empowering the youth to contribute to making a difference in their community through active engagement in meaningful decision-making [2].

Although photovoice has been predominantly used as a research methodology, some researchers have explored its use as a pedagogical strategy [5–7]. We position our use of photovoice within this student-led participatory research endeavor as a research approach and a transformative pedagogical tool. For the undergraduate students involved, this meant learning from each other and from facilitating the research process, as well as learning from the co-facilitator participants involved in the study. Research strategies such as photovoice that immerse students in the lived experiences of a local community and engage them in critical thinking are one of the ways to understand social issues faced by marginalized communities [8]. In the parent study, students facilitated a photovoice study that explored the socially and culturally constructed experiences of the COVID-19 pandemic among BIPOC older adults and frontline healthcare workers [9]. In the current work, we aimed to capture the various student experiences facilitating the photovoice research project with BIPOC older adults and frontline healthcare workers.

There is limited evidence of undergraduate students facilitating photovoice research projects. The photovoice literature has focused mainly on research collaborations between researchers in academia and community partners [10,11]. Photovoice pedagogy has been used to foster educational values such as inclusion, diversity [3], and appreciation for science [12,13]. Common reported outcomes of these studies indicated that photovoice pedagogy increased students’ critical self-awareness [5,14], sense of autonomy, and empowerment as they became more active and responsible for their own learning [15,16]. Undergraduate student participation in a photovoice research process can benefit students and their community partners [17]. There is evidence that undergraduate students’ participation in the CBPR research process can help them learn and apply appropriate research methods and good communication skills [18] and empower them to engage with local community members to create change [19]. Other studies show that such engagement can encourage autonomy, purposeful task orientation, student reflection, and close connections with the local community [20,21].

### 1.1. Photovoice as a Transformative Pedagogical Strategy

Many students come to colleges and universities with well-established views about the world and hold many assumptions, limitations, and misconceptions that may be hard to transform and usually act as an added challenge when they are not provided the opportunities to integrate the academic and their personal views together [22,23]. The notion of transformative learning provides the basis for this study. According to transformative learning theory, individuals improve their understanding of the world by revising their habitual ways of thinking, feeling, and acting (frames of reference) and specific attitudes, values, beliefs, or judgments (points of view) [24]. Three concepts of transformative learning theory identified by this study [25] include (1) experiencing a disorienting dilemma (kind of experience that is challenging or painful), (2) critical reflection, and (3) perspective transformation. The core of transformative learning theory is that learning in adulthood should be transformative if educators present the learners with disorienting dilemmas that are intellectually and emotionally provocative to allow them to critically reflect and re-examine their existing values, views, and knowledge [26,27]. The individuals’ engagement in self-critical reflection on their own experiences can allow room for transformation and expansion of one’s own thoughts, feelings, and actions and could bring about the transformation of a frame of reference that reorients the habit of minds and, subsequently, the points of view [28]. The concept of critical reflection in transformative learning aims to increase the individual’s awareness to examine their world closely to be able to re-see it through the lens of the new learning [24]. Like transformative learning, photovoice emphasizes individuals’ central role in re-exploring their world and reframing it through their active participation in redefining that world [15,29,30]. As a transformative pedagogical strategy, photovoice empowers students to critically examine their beliefs, values, and knowledge, appreciate multiple perspectives, and develop a sense of critical consciousness and agency [31]. One previous study [3] argued that photovoice as a pedagogy could effectively provide a compelling, critically reflective experience wherein students think and rethink their engagements with self and others.

### 1.2. The Current Study

Numerous studies have reported on college student participation in photovoice research projects to explore various social issues [2,4]. A few studies have documented the experiences and reflections of graduate students as facilitators of photovoice research [6,30]. To our knowledge, no study has yet reported on the reflections of college students who facilitated a photovoice research project. In this study, undergraduate students were recruited and trained to be photovoice project facilitators rather than participants. As such, we posit that having college students facilitate a photovoice research study may help them critically examine their knowledge and perception of the lived experiences of their study participants during the COVID-19 pandemic. The study aimed to answer the following research questions: (1) What are college students’ initial impressions of BIPOC older adults and frontline healthcare workers who participated in the photovoice research study? (2) How does facilitating a photovoice research project influence college students’ perception and knowledge of BIPOC older adults and frontline healthcare workers during the COVID-19 pandemic? (3) What are the benefits of college students facilitating a photovoice research project with BIPOC older adults and frontline healthcare workers?

## 2. Materials and Methods

### 2.1. Design

This descriptive qualitative photovoice study was conducted between January and May 2022. The photovoice research process allowed for the active involvement of student facilitators with participants in a way that was relevant and meaningful to the students.

### 2.2. Recruitment of Participants

Upon receiving the Institutional Review Board (IRB) approval (Protocol number: #1624), the principal investigators recruited junior and senior undergraduate research assistants at a small liberal arts Catholic women’s university in the Midwest for the photovoice research project. Students were recruited from the “Katies for Aging Research and Equity (KARE)” program on campus and the undergraduate public health program via email and personal contact. Students were included in the study if they were current undergraduate students and met the National Institutes of Health (NIH) eligibility criteria of being minority underrepresented in biomedical, clinical, behavioral, and social sciences research.

### 2.3. Training of Student Facilitators (SFs)

The investigators conducted a three-day training of 13 student facilitators (SFs). During the training, the researchers explained the study purpose, the photovoice methodology, the use of the camera, and ethical photography. This training was to prepare the SFs to work with Black, Indigenous, and persons of color (BIPOC) older adults (OAs) and frontline healthcare workers (FLHWs) in the photovoice research project. Details of the training are shown in Table 1.

After the training, 5 of the 13 students dropped out of the study due to hectic class schedules or personal reasons. Eight SFs participated in the study. Table 2 displays the demographic characteristics of the SFs.

To ensure consistency in training, the SFs were asked to roleplay each section of the training, followed by investigators’ feedback and a general discussion with the entire student group. The roleplay activity provided real-world scenarios to help students learn and opportunities for them to critically observe their peers. After the training, the investigators paired eight student facilitators with either an OA and/or an FLHW for the photovoice research project based on their availability and academic schedule. They met weekly with the students to reinforce the photovoice concepts and process. The students were charged to ask the participants to take photos of people, situations, or objects that reflect any challenges they faced during the COVID-19 pandemic and how they sought for meaning during those challenges. Eight older adults and five frontline healthcare workers participated in the study. The photovoice research was conducted in four stages.

### 2.4. Procedure

#### 2.4.1. Introduction of Photovoice Methodology

Each SF contacted their assigned participant via phone and set a meeting date. During the first meeting, the SF explained the purpose of the research study and questions, the photovoice methodology, the risks and benefits of participation, and the principles of ethical research. Each SF reviewed and checked for the participant’s understanding of the informed consent process before obtaining consent. In the second meeting, the SFs demonstrated how to use the camera, explained ethical photography, and had the participants practice using the camera, including troubleshooting. The SFs reminded each participant to obtain a waiver from any individual in their photographs.

#### 2.4.2. Taking the Photos

The SFs provided digital cameras to participants. Some participants were allowed to use their phone cameras to accommodate their preferences. The SFs gave their participants one week to take photos. They asked the participants to take photos of (1) people, situations, or objects that reflect any challenges they faced during the COVID-19 pandemic and (2) photos of people, situations, or objects that reflect how they sought to find meaning during those challenges. During the week of photography, the SFs remained in contact with their matched participant(s) via a phone call. They identified and met the level of support the participants needed and provided verbal and written reminders of the intended focus of the photographs.

#### 2.4.3. Photo Selection Process

Once participants finished taking photos, each SF conducted and recorded a semi-structured interview. The participant was shown their photographs on a laptop or tablet and asked to choose two photos of their challenges during the COVID-19 pandemic and two photos of how they found meaning during those challenges. To help narrow down the most relevant photos, research assistants asked the participants questions such as “why did you take the photo?”, “how did you feel during the process?”, “what does the photo represent?” and “how does this answer the research question?” posed in the parent study [9]. Once two photos representing challenges and meaning were selected, the student facilitators were present to technically support participants while they wrote their narrative for each photo. To document the participant’s narrative of the images, the SFs used the SHOWeD technique asking each participant the following: (1) What do you See in the picture? (2) What Happened, or is Happening, in the picture? (3) How does this relate to Our lives? (4) Why does the problem, concern, or strength exist? (5) What can we Do about it [1]? SFs created files with the selected photographs and narratives and recorded the photo selection and narrative process on a shared protected Box account on their computers. The investigators had access to these shared folders.

#### 2.4.4. Facilitation of Focus Group Discussion

The investigators and the SFs conducted two online focus group sessions with older adults and one with frontline healthcare workers. One older adult focus group session was conducted in Spanish. The investigators asked two SFs to facilitate each focus group discussion. During the focus group sessions, the SFs asked each participant to share and discuss two images respectively of the challenges they faced and how they found meaning during those challenges. The SFs asked other focus group members to reflect and share their views on the images and narratives their fellow participants shared. This guided discussion was intended to deepen individual narratives of photographs. The virtual focus group discussion sessions were recorded on Zoom. Having SFs conduct semi-structured interviews and focus-group discussions with participants was intended to broaden their perspectives about their experience with participants and the research process. The SFs were paid for their time facilitating the photovoice research activities.

### 2.5. Data Collection and Analysis

Throughout the study, the investigators asked the students to critically reflect and write on the various aspects of their experience, facilitating the photovoice research activities based on weekly structured reflection questions. Examples of such questions include the following: (1) Describe your impression and experience during the initial photovoice session with your assigned participant(s). (2) How have your understanding and perception of the participants changed due to facilitating this photovoice research study? (3) What lessons did you learn, and how have you benefited from working with BIPOC older adults and frontline healthcare workers? (4) What is your perception of the usefulness of photovoice in understanding the struggles of BIPOC older adults and frontline healthcare workers? Students were also encouraged to write about other issues/situations they experienced during the process.

The investigators conducted a thematic analysis of students’ weekly written structured reflections following the six steps described in this study [32]. First, the research team, consisting of one investigator and four research assistants, met and familiarized themselves with the data by reading and rereading each written response. We reviewed and followed each student’s weekly reflections, which allowed for examining change trajectories within individual students over time. Each student was assigned a pseudonym (SF) and a number, e.g., SF1, SF2, etc. Second, the research team independently coded all responses to each reflection question and agreed on 96% of the initial coding instances. We reviewed, discussed, and resolved the remaining differences in the coding. Third, the research team placed similar codes together to create potential themes. During this data analysis step, the team worked collaboratively and frequently had to revisit codes from Step 2 and the original data from Step 1. Fourth, the team looked for potential overlaps, similarities, and differences in how the SFs responded to each question. Fifth, themes were defined and named. Throughout the data analysis process, the team went back and forth between the themes, codes, and participant responses to ensure that they accurately organized the codes. The research team noted examples of SFs’ responses under each theme. Finally, the research team included individual participant quotes as examples in the results section.

## 3. Results

Students’ written reflections highlighted their perception of and experience working with BIPOC older adults and frontline healthcare workers in the photovoice research project. Three themes emerged from the analysis of their reflection: (1) impressions of BIPOC older adults and frontline healthcare workers; (2) lessons learned from the stories of BIPOC older adults and frontline healthcare workers; and (3) outcomes of facilitating a photovoice research project.

### 3.1. SFs Impression of BIPOC Older Adults and Frontline Healthcare Workers

The SFs described the BIPOC frontline healthcare workers as friendly, pleasant, and easy to talk to. One SF recalled how she bonded pretty quickly with her participant because they are both public health majors and have worked in healthcare. The SFs’ overall impression of BIPOC frontline workers who were also college students is that they were selfless and resilient. The SFs expressed great admiration for their hard work during the COVID-19 pandemic and empathy for their challenges of balancing work in care homes and school responsibilities. Some responses include the following:

My initial impression of BIPOC frontline healthcare workers is how resilient they are and the strength they possess to do their work. It is a tough job, and they persevered. [SF6]

She is stretched very thin. She has a lot of classes that she is taking, and she works almost every day in the care home. [SF8]

The SFs had similar impressions of BIPOC older adults, who they described as “kind,” “inspiring,” “flexible,” “resilient,” and “hardworking.”. They were inspired by how their participants sought and found meaning amidst life challenges.

BIPOC older adults are hardworking and resilient. Many of them went through difficult times working long hours so that their children could live a better life. Those who are first-generation immigrants went through an especially difficult time adjusting to a new country, language, and customs, oftentimes without many family members around. [SF5]

My initial impression of the BIPOC older adult I met with was that she was very kind. Not only this, but she is a woman who has found meaning in many areas of her life. She is an activist, a grandmother, a teacher, and so much more. I enjoyed talking with her and figuring everything out alongside her. It was so inspiring to meet someone with so much experience and life and to feel like I was connecting and making some sort of positive impact on her and my own life. [SF2]

Despite having an abusive and hard past, one older adult mentioned that she continues volunteering at community events and keeping activism as part of her main purpose.

The SFs also noted the interest and eagerness of BIPOC older adults to participate in the study to share the stories of their struggles during the COVID-19 pandemic. According to one SF, her participant “expressed how excited she is to be a part of this research and happy that she is participating.” [SF4]. Another SF wrote: “My older adult participant mentioned that she always longs to meet new people and share her stories.” [SF1] It is evident that this research provided the opportunity that would not have been available to these participants to share stories of their experiences during the COVID-19 pandemic and how they dealt with the challenges.

### 3.2. Lessons Learned from the Stories of BIPOC Older Adults and Frontline Healthcare Workers

The SFs were paired with BIPOC older adults and frontline healthcare workers to learn about their lived experiences during the COVID-19 pandemic. The reflections of the student facilitators showed that BIPOC frontline healthcare workers felt mistreated, undervalued, and not appreciated for their work. A few of them narrated their experiences of microaggressions at their workplaces during the pandemic. The SFs wrote that some BIPOC frontline healthcare workers also carry significant emotional burdens and have a minimal support system.

I learned that they carry a greater emotional burden, felt undervalued, and underappreciated now than before the pandemic. Being a Certified Nursing Assistant (CNA) is already a physically demanding job. However, during the pandemic, they discussed the difficulties they encountered with their patients refusing to wear masks, including the racial microaggressions they experienced in the workplace. [SF5]

I learned that BIPOC frontline workers are somewhat mistreated at work and not having the support system they need. The first participant that I had gave me so many scenarios of racial comments made to her by long-term care residents and microaggressions. [SF7]

Regarding BIPOC older adults, the SFs learned that they felt isolated, were afraid of getting sick with COVID-19, and face additional stressors such as language barriers when seeking medical help, lack of transportation, and the inability to send money to their loved ones in their home country.

I realized how she and other older adults faced many more invisible challenges relating to isolation and fear during the pandemic that I was unaware of. Participating in this study opened my eyes to the unique challenges my participant faced as an older adult and nurse during the pandemic. [SF5]

My participant described that during the COVID-19 pandemic, the feeling of isolation was very present. As we know, COVID-19 had many negative impacts on our society. However, it is even more evident that BIPOC individuals face additional stressors. Such as language barriers when seeking medical help and transportation and the inability to send money back to their home country. [SF4]

Despite their difficult situations, the SFs noted that BIPOC older adults derived a sense of meaning and purpose by being socially engaged in their communities.

### 3.3. Outcomes of Facilitating a Photovoice Research Project

In individual reflections, the SFs examined the applicability of photovoice in increasing their (1) awareness of the struggles of BIPOC communities during the COVID-19 pandemic, (2) critical self-awareness, (3) sense of empowerment, and (4) improved research skills.

#### 3.3.1. Increased Awareness of the Struggles of Communities of Color

Analysis of their reflections revealed that SFs overwhelmingly acknowledged the effectiveness of the photovoice methodology in allowing multiple perspectives on the same issue and providing opportunities to capture the struggles and raw experiences of BIPOC older adults and frontline healthcare workers during the COVID-19 pandemic and their sources of resilience.

Photovoice is an incredibly meaningful way to understand the impact racism, isolation, and the pandemic overall have had on BIPOC older adults. However, pictures only show so much, which is why it is amazing that there are narratives that go along with each one! It allows many voices to be heard regarding the same topic. [SF2]

My admiration of BIPOC healthcare workers has increased because I now know more about the additional challenges they face in the workplace, which range from microaggressions to unequal treatment from management/coworkers to the struggles of working in a healthcare setting during a pandemic. Despite all the added challenges they face, they continue to show up to work daily to fulfill their duties and offer a helping hand. [SF5]

Photovoice provides a wonderful way of viewing what gives hope to those who are BIPOC. Through their beautiful photos, we can see the kind of hope that helped them continue to push through. [SF4]

#### 3.3.2. Increased Critical Self-Awareness

Facilitating the photovoice research allowed the SFs to deeply think about the stories narrated by BIPOC healthcare workers about the unfair compensation for their work “Now, hearing the personal stories of a BIPOC frontline healthcare worker has really challenged my thinking of how their work is compensated and appreciated by (their bosses and) healthcare stakeholders.” [SF6] They then echoed the need to combat pay inequities through equitable wages for people of color in frontlines. The SFs also appreciated the connection they could make between research and real-life situations. One SF remarked the following:

Being a part of this photovoice project has allowed me to meet multiple different older adult BIPOC individuals with lifelong experiences and helped me really connect the dots between what I have been learning and what interacting with older adults themselves is like. [SF2]

One student facilitator discussed how her participation in the photovoice improved her recognition of the importance of treating older adults just like anybody else “with equitable care, of course”!

#### 3.3.3. Increased Sense of Empowerment

Facilitating the photovoice project ignited the students’ interest in working with BIPOC older adults and bridging the intergenerational gap between young people and older adults, as well as reducing health disparities experienced by communities of color in their future professional practice.

This study has greatly encouraged my interest in working with BIPOC older adults in the future! Older adults are still so misunderstood by the general population; I am motivated to work to bridge the age divides between the young and old as best as I can. [SF2]

It has influenced me in a good way, I have always been intrigued with sociology, and I am pursuing public health due to the health disparities we see in our society. This study has added a new lens and helped me not exclude this population when considering preventative measures in public health. [SF4]

#### 3.3.4. Improved Research Skills

Through their participation in the photovoice research process, the SFs improved their critical thinking skills, leadership and collaborative skills, and gained confidence in explaining the photovoice research process to participants and an understanding of the importance of ethics for the conduct of research. For example, one student remarked the following:

I learned how to communicate with my participants, coach the participants on taking pictures, how to select photos, and what we are looking for in a narrative. [RA8]

## 4. Discussion

This study presents the reflections of college students who facilitated a photovoice research project with BIPOC older adults and frontline healthcare workers during the COVID-19 pandemic. In this study, we trained college students in photovoice methodological skills by introducing photovoice as a participatory qualitative methodology, explained ethics issues and the informed consent process, and clarified the steps and requirements for the photovoice project. Students were afterward paired with BIPOC older adults and frontline healthcare workers. They facilitated the photovoice research project in four stages, regularly meeting with their participants in small groups during the week to discuss what their photographs meant to them and how the photographs reflected their experiences of racism, isolation, and meaning. Details of the findings of the parent study can be found in this study [9]. Throughout the project, the undergraduate students engaged in an introspective process of reflecting on their learning. In their reflection journal, students shared their impressions about BIPOC older adults and frontline healthcare workers; lessons learned from the stories shared by participants; and the outcomes of facilitating a photovoice research project with communities of color.

In their weekly journal, student facilitators commented on how both older and younger BIPOC participants were friendly and kind to them, flexible with the research activity scheduling, inspiring with their stories, and selfless. Students’ reflections indicated that their participants experienced greater emotional and psychological burdens during the COVID-19 pandemic, likely exacerbated by their experience of systemic racism. Student facilitators overwhelmingly recognized participants’ resilience and perseverance despite these life challenges. Resilience and perseverance in the face of adversity have been noted as essential for human flourishing across various populations, settings, and the human life span [33]. Students noted from the stories that BIPOC older adults derived a sense of life meaning and purpose through active engagement in their community by volunteering at community events and participating in social activism. Our results are consistent with previous studies, which found that communities of color always find ways to recover from adversities [34] by building strong social networks [35,36] and having a sense of personal meaning [37,38].

Students further learned from the stories of BIPOC frontline healthcare workers that they felt underappreciated and undervalued by their bosses as illustrated by the following comment from a frontline healthcare worker: “Most of us are overworked and sometimes we don’t get recognition from our managers or supervisors...” The student facilitators felt that BIPOC frontline healthcare workers were risking their lives to care for others yet were undervalued. This view confirms the work of the parent study that showed that BIPOC frontline healthcare workers reported feeling disrespected, unappreciated, and undervalued by their employers [9]. Interestingly, BIPOC older adults appreciated the opportunity to participate in the research to share their stories of struggles and how they found meaning. The importance of storytelling as an approach to developing resilience has been highlighted in several studies [39,40]. Having college students listen to the stories of their BIPOC participants could be an effective way for both the storytellers and their listeners to find meaning in events, transmit emotion along with information, and build empathy and compassion [41]. The stories of BIPOC participants can provide models of strength and empowerment and could help college students validate their own identities to the self and the world. Students not only found their stories inspiring but developed interest in bridging the intergenerational gap between young people and older adults and fighting to reduce inequities in their future professional practice. Although each BIPOC participant had their own unique story, all the student facilitators commented on the experience of racism, microaggression, and discrimination shared by each participant.

This project allowed the student facilitators to gain a deeper insight into income inequality in society, particularly for the BIPOC communities. Students remarked in their reflections that without their direct involvement in this photovoice research process, they would not have known first-hand the deep struggles faced by BIPOC older adults and frontline healthcare workers during the pandemic. This finding is surprising with regard to BIPOC older adults knowing that the majority of the student facilitators have parents who are BIPOC themselves and so must have witnessed their parents struggle during the pandemic. Students wrote that their involvement in the photovoice project increased their interest not only in working with communities of color but fighting to reduce systemic inequities experienced by these communities. Our findings highlight the importance of photovoice in raising one’s sense of critical consciousness about social issues and society [1,24,30,31] and improving college students’ understanding of the struggles of the local communities [42]. Students learned meaningful life lessons from their participants, and such a shared body of knowledge can promote student personal growth [24,43]. Moreover, students improved their critical thinking and creative skills, acquired some qualitative research skills, leadership, and collaborative skills, and came to understand the procedure and importance of ethical conduct of research. Our finding corroborates the results of previous studies which reported on the usefulness of photovoice methodology in developing and strengthening qualitative research skills and stimulating critical thinking, creativity, and problem-solving skills among college students [44,45]

Finally, the findings of this study support transformative learning theory [22,24], which emphasizes the role of critical self-reflection in increasing individuals’ awareness of their world, so they are able to re-see it through the lens of the new learning. Students reflected critically on their experience working with participants throughout the photovoice research project. There is evidence that engagement in such reflections can expose an individual to different viewpoints and perspectives on an issue, which in turn becomes the basis for a person’s attitudinal or behavioral transformation [46].

### 4.1. Implications for Practice

Students raised concerns regarding the experience of workplace mistreatment that BIPOC frontline healthcare workers shared with them. This finding will help to highlight the inequity and bias that BIPOC frontline healthcare workers experience in their various places of work. We suggest and encourage hospital/long-term care managers to develop diversity, equity, and inclusion (DEI) training for their employees and patients/clients. Such training will help foster awareness and understanding of how people with different backgrounds can work together harmoniously. Student reflections highlighted the difficulties that participants who are first-generation immigrants go through adjusting to a new country, language, and customs, hence the need for policymakers/local governments to create programs that support cultural identity, heritage sharing, and rapport building within immigrant and minority communities for better integration into the receiving society.

### 4.2. Limitations

Our study has some limitations. First, our study was conducted with a sample of female students from an all-women medium-sized university in the Midwestern United States. The experience of these students with BIPOC older adults and frontline healthcare workers may be unique and not generalizable to the larger population. Hence, we suggest replicating the findings in co-educational colleges and universities to fully capture the wide range of experiences and perceptions of younger adults toward people of color. Second, our sample size was small (*n* = 8); a larger sample size could yield more diverse feedback and generalizable results. We did not explore differences in the viewpoints of BIPOC student facilitators against their non-BIPOC student counterparts. However, a comparison would not have made a significant difference in the findings due to the small sample size.

## 5. Conclusions

This qualitative study aimed to explore students’ perceptions and experiences facilitating a photovoice project with BIPOC older adults and frontline healthcare workers. The results added to the literature of both transformative learning and photovoice pedagogy and could be a resource for adult educators who consider research for support in their pedagogical approaches when working with college students. The study found the photovoice methodology very useful in understanding the lived experiences of underserved communities during the COVID-19 pandemic while increasing the SFs’ critical self-awareness, sense of empowerment, and research skills. The photovoice project as a pedagogical strategy allowed the research team to gain insights into student experiences by enabling them to reflect on their experience working with the participants. The hands-on experience of facilitating photovoice research and the grassroots engagement of students with BIPOC participants may have helped prepare them work for policy change in their future professional practice. Hence, researchers should strive to actively engage college students in research with underserved communities as they could serve as a voice to elevate the concerns of local communities.

## Figures and Tables

**Table 1.** Training sessions with student facilitators.

Session	Content	Objectives	Assessment
Day 1: 3 h	Study overview, research ethics	Describe the study rationale of the study, purpose, and issues involving human subject research. Explain key aspects of participant engagement, retention, and relationship. Explain ethical photography.	Group discussion Written critical reflection
Day 2: 3 h	Photovoice research technique	Explain the photovoice research technique. Discuss the implementation steps. Demonstrate proficiency in describing the process and tasks to others.	Group discussion Written critical reflection Roleplay
Day 3: 3 h	Myth busting, camera use, and troubleshooting	Explain some misconceptions about aging and working with older adults. Practice using the camera, including troubleshooting.	Group discussion Roleplay Written critical reflection

**Table 2.** Demographic description of student facilitators (*n* = 8).

Demographic Description	*n* (%)
Gender	
Female	8 (100)

Class rank	
Sophomore	1 (12.5)
Junior	4 (50.0)
Senior	3 (37.5)

Major	
Public Health	3 (37.5)
Psychology	2 (25.0)
Biology	2 (25.0)
Exercise and Sport Science	1 (12.5)

Race/Ethnicity	
Black or African American	2 (25.0)
White or Caucasian	2 (25.0)
Hispanic or Latino	2 (25.0)
Asian	2 (25.0)

KARE Scholar	
Yes	3 (37.5)
No	5 (62.5)

Language	
English only	5 (62.5)
Spanish and English	2 (25.0)
Somali and English	1 (12.5)

## Data Availability

The data presented in this study are available on request from the corresponding author. The data are not publicly available due to privacy restrictions.
